# This Striking Blue Made Pigment History. Could Red Be Next?

**DOI:** 10.1021/acscentsci.5c01396

**Published:** 2025-08-11

**Authors:** Carrie Arnold

## Abstract

Mas Subramanian’s hunt to create red that’s vivid, durable, and safe.

## Graphical abstract

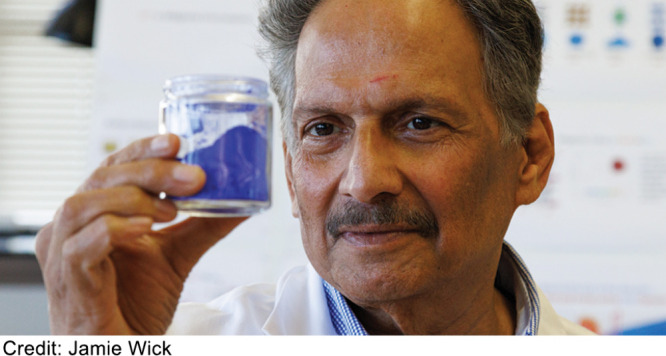

Lightning first struck Mas Subramanian over 15 years ago. When he and his graduate student Andrew Smith put a mixture of rather mundane powders into the lab oven at Oregon State University, the pair wanted to discover exotic new metal combinations to improve supercomputing. What they pulled out of the oven, however, wasn’t that.

**Figure:**
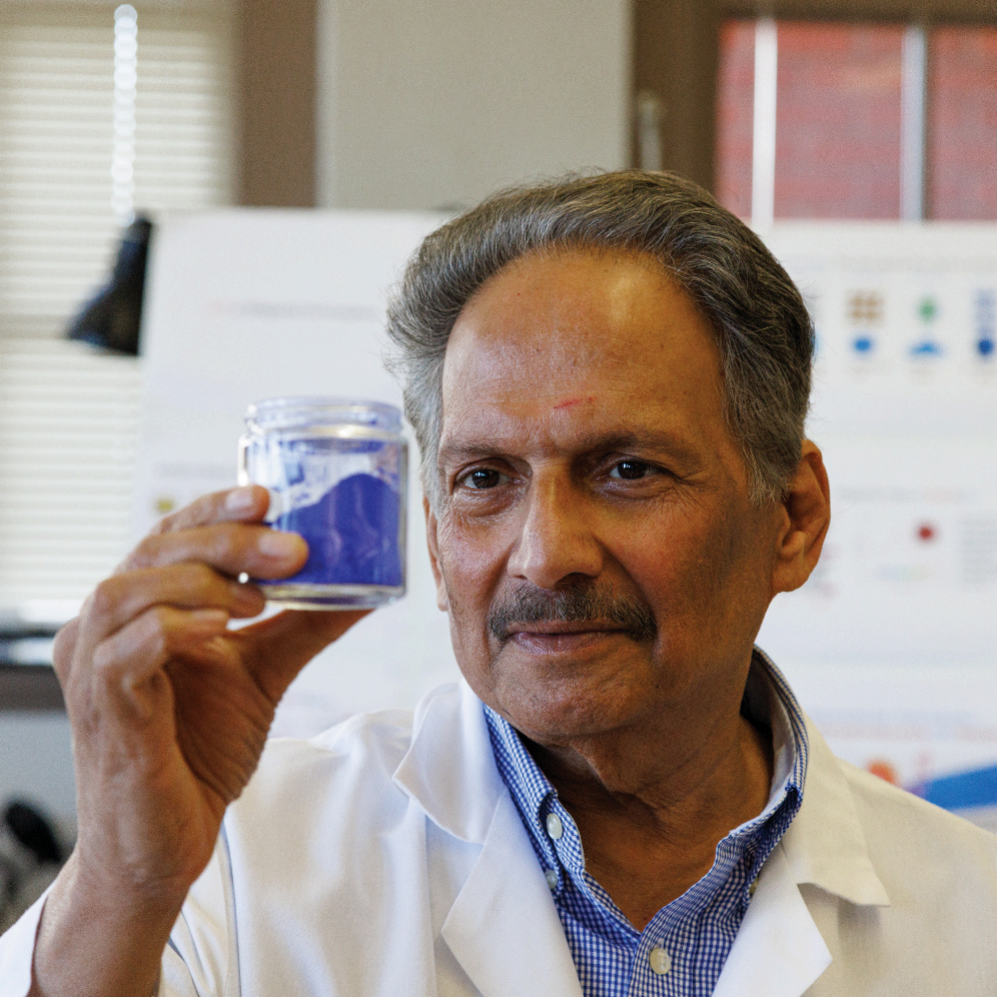
Mas Subramanian at Oregon State University earlier this year with his YInMn blue. Credit: Jamie Wick.

By doping manganese into yttrium indium oxide to create YIn_1–*x*_Mn_*x*_O_3_, Smith and Subramanian had cooked up a pigment so blue that adjectives like azure, cerulean, and cobalt felt like understatements.

“I was so shocked to see this beautiful blue come out,” Subramanian says.

Subramanian had created the first new synthetic blue pigment since cobalt blue two centuries before. The chemist called the new pigment YInMn (pronounced “yin-min”) blue. Crayola named the shade Bluetiful and offered a special edition crayon inspired by the pigment. The project opened Subramanian’s eyes to the possibility of rational pigment design—creating new colors using the targeted and deliberate manipulation of chemical structure and function.

In the years since his initial discovery, Subramanian has tinkered his way through most of the rainbow by adding various metals to the original yttrium oxides to make yellow, orange, fuchsia, violet, and lime green. One color, however, has continued to elude Subramanian. “Nobody knows exactly how to create a new red pigment,” he says.

Of course, plenty of red pigments already exist, but they all have their drawbacks. Organic reds can be difficult to work with and will fade over time. Inorganic reds might be lightfast but many, such as cadmium red and vermilion, are also toxic.

The problem isn’t just intellectually worthwhile; it’s also very lucrative. Specific hues like rosso corsa, the Italian racing red, or Tiffany Blue have become iconic in their own right. The global pigment market was worth an estimated $44.68 billion in 2024, according to Straits Research.

Finding a red that is permanent, safe, and inexpensive has become Subramanian’s new challenge. Last year, he used a divalent chromium oxide found in lunar minerals as inspiration for a magenta pigment. It’s not red, he admits, but he’s getting closer.

**Figure:**
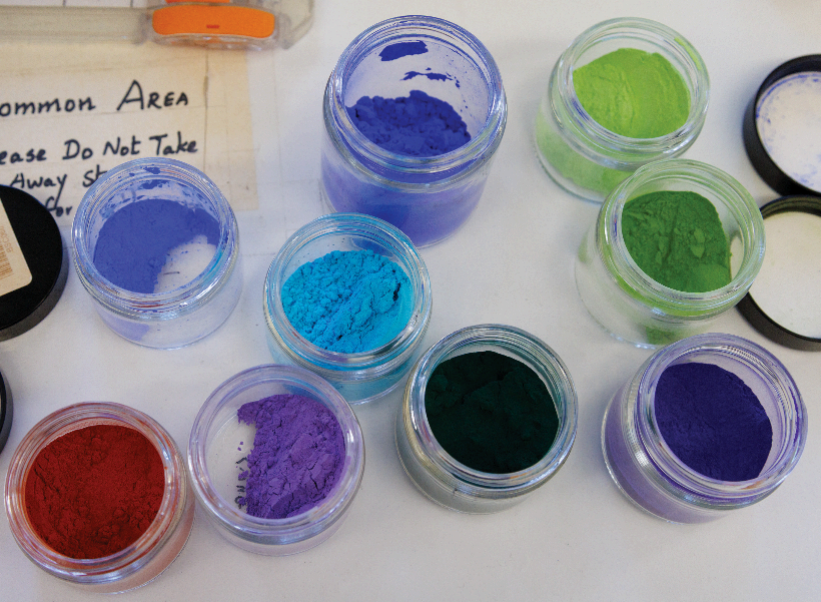
Mas Subramanian wants to design a full artist’s palette of pigments. Credit: Jamie Wick.

## Color as culture

Ever since humans first began painting on the walls of caves, we have searched for pigments to color our art, walls, bodies, and pottery. For millennia, the natural world was our only source of dyes and pigments, and nature offered a huge variety of hues to work with. Charcoal, cochineal, indigo, and Tyrian purple all populated the rainbow of colors. These organic dyes could be dissolved in solvents, which made them relatively easy to work with, especially for coloring thread and cloth.

But natural colors had their limitations: the color and its intensity varied from batch to batch, the dyes faded over time, and many compounds were eye-wateringly expensive.

Inorganic pigments could also be used as colorants for pottery, paints, and other items. Here, too, expense was an issue, as many inorganic pigments were created by crushing semiprecious gemstones, such as the prized deep blue pigment ultramarine, which was made from lapis lazuli.

People in ancient Egypt created the first synthetic pigment—Egyptian blue is a copper calcium silicate (CaCuSi_4_O_10_ or CaO·CuO·4SiO_2_) that the Romans called caeruleum—but the field didn’t really take off until the Industrial Revolution. Chemists experimenting with coal and petroleum by-products found themselves with a range of organic dyes, including mauve-colored aniline dyes (the original mauve being a serendipitous discovery by William Henry Perkin in 1856). Advances in inorganic chemistry heralded a range of synthetic pigments, such as Prussian and cobalt blues (Fe_4_[Fe­(CN)_6_]_3_ and CoAl_2_O_4_, respectively), vermilion (HgS), and primrose yellow (BiVO_4_).

Subramanian learned this pigment chemistry as an undergraduate student at the University of Madras. But his long fascination with crystal structures and the work of Linus Pauling led Subramanian to focus his attention on solid-state chemistry for his doctorate at the Indian Institute of Technology Madras.

Subramanian spent 22 years at DuPont’s headquarters, where he worked on everything from superconductors and thermoelectrics to catalysts and organic synthesis. But a call to teach always pulled at him, and in 2006, he left DuPont and moved to Oregon State University, where he returned to his previous interest in creating novel materials to improve computers. Which is how, in 2008, Subramanian and PhD student Smith found themselves staring at YInMn blue.

“Blue pigments are always desired, and there are some on the market, but all these have some disadvantages,” says Gerhard Pfaff, a retired materials scientist from the Technical University of Darmstadt. “That’s why the development of YInMn blue was so important.”

The 2009 *Journal of the American Chemical Society* paper describing the new color grabbed headlines around the world. The paper detailed how trivalent manganese (Mn^3+^) nestled in a trigonal bipyramidal lattice of yttrium and indium could produce one of the most vibrant blues the world had ever seen. Further research showed that YInMn strongly reflected near-infrared radiation and was heat and light resistant and nontoxic. These properties made the compound useful in a world increasingly affected by climate change and toxic pollution.

Blue became a theme in Subramanian’s life, coloring the single-serve coffee maker in his office, his checkered shirt, and his navy slacks. Smith and Subramanian patented their discovery and licensed YInMn blue to Shepherd Color in 2015. Subramanian’s wife, Rajeevi, a chemist and watercolor artist, fell in love with the color.

The transition metals required to make YInMn blue, however, mean that the color will never be cheap. Artists not married to Subramanian will need to fork over upward of $50 for a thumb-sized tube of YInMn blue watercolor paint (I snagged a tube in a Black Friday sale for $35; other pigments of the same brand ran $15–20 for the same size and quality).

“It’s a very nice blue, but I hesitate to say it’s a good pigment. Why? Because it has very expensive raw materials,” Pfaff says.

## A rational red?

In 2009, Subramanian began tinkering with the YInMn stoichiometry, adding and subtracting amounts of the original three transition metals as well as doping the mixture with iron, copper, indium, or titanium. The range of hues seemed nearly endless. Except for one tricky hue.

Despite all his work, Subramanian has never been able to make a true red pigment. In one sense, Subramanian’s task is straightforward: all he needs to do is find a compound that will reflect light waves between 620 and 750 nm. But the challenge is not just finding the compound—Subramanian knows that, given enough time and money and luck, he will eventually stumble his way onto a novel red pigment. Rather the goal is understanding the inner workings of pigments so that he can truly design them.

Unlike in the field of drug discovery, where the deliberate selection of functional groups and other chemistry can give the resulting compound its desired activity, rational design has yet to hit the world of pigments. That’s partly due to the sheer number of interacting variables that determine a pigment’s final coloration, including a metal’s oxidation state, its neighbors in the crystalline lattice, and how all the atoms are bonded together.

“It’s pure solid-state chemistry to try to design a complex atomic structure like a pigment,” says Manuel Gaudon, an expert in inorganic pigments at the Institute of Condensed Matter Chemistry of Bordeaux. “To get something not so explored by the other literature, you have to use some exotic parameters to try and get new effects.”

“Nobody was rationally able to sit and say, ‘This is the component that produces blue,’” Subramanian says. But his years of work on YInMn blue gave him a hint that the Mn^3+^ swaddled by yttrium and indium was responsible for the pigment’s eye-popping color.

There may be more to the blue of YInMn, however. “Color is not given by only one factor,” Gaudon says. “There are a lot of parameters to control.”

For many companies, the challenge of creating new pigments has meant that organizations have shifted their attention to optimizing and tuning existing pigments.

“The key improvements are centered on optimizing colorimetric properties and increasing resistance to various environmental and chemical conditions. This includes resistance to aggressive chemical media such as acids and bases, as well as improved lightfastness, weather resistance, and thermal stability,” says Anne Stephens, global vice president of R&D for color solutions at Vibrantz Technologies, a company that makes high-performance colorants and coatings, in an email.

In his search for red, Subramanian has turned his attention away from this planet and toward lunar materials.

On Earth, chromium exists in a range of oxidation states, from the very stable, highly toxic hexavalent chromium to the less stable but far safer trivalent chromium used in a range of green and yellow pigments. Lunar rocks, however, contain trace amounts of oxides containing divalent chromium (Cr^2+^). Subramanian and his graduate students immediately realized that these lunar chromium oxides contained the same number of unpaired electrons as the trivalent manganese at the heart of YInMn blue. They were inspired.

Their resulting creation effectively replaces the divalent copper used in Egyptian blue with a divalent chromium. The material has a rose-red color that is nontoxic and reflects sunlight.

**Figure:**
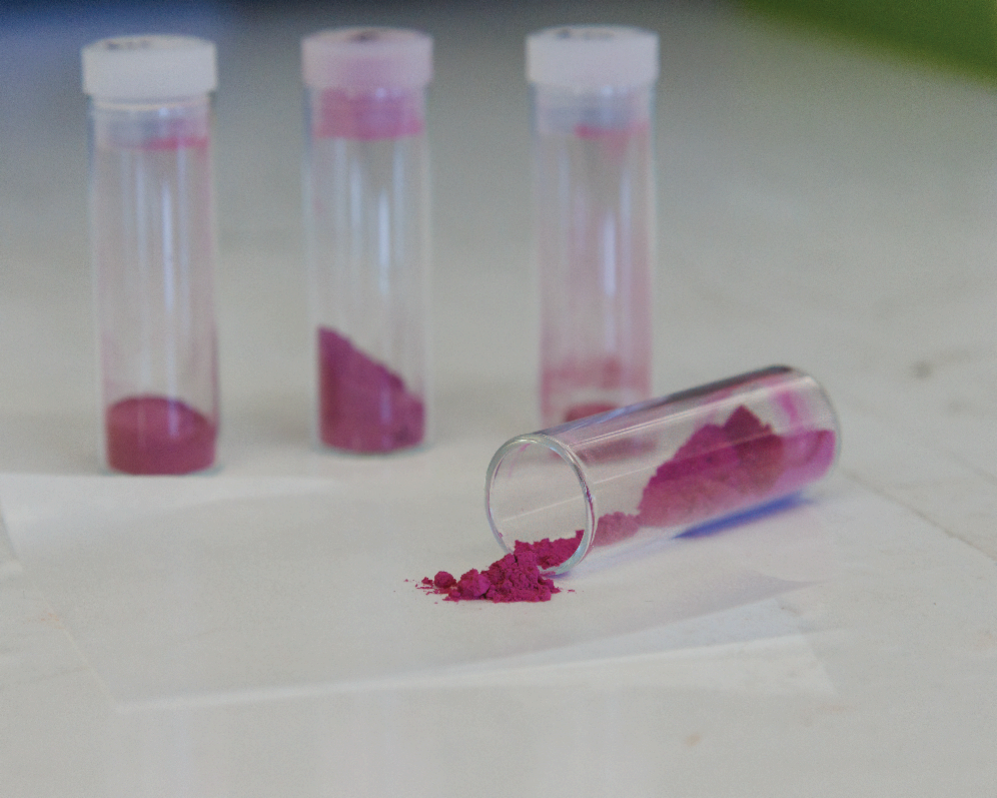
Mas Subramanian’s new durable, reddish magenta is inspired by lunar mineralogy. Credit: Jamie Wick.

It is not the red of Ferraris or stop signs, but for Subramanian, the moon-inspired magenta represents a huge step forward for rational pigment design. With magenta under his belt, Subramanian has returned to the lab to see if tweaking the components of the divalent chromium oxide can yield a true red.

“It’s not easy to predict everything. We don’t know how to create a perfect structure without going to the lab and mixing it and seeing whether it works or not,” Subramanian says.

*Carrie Arnold is a freelance contributor to* Chemical & Engineering News, *an independent news publication of the American Chemical Society. A version of this story appeared in C&EN.*

